# Risk, Benefit, and Moderators of the Affect Heuristic in a Widespread Unlawful Activity: Evidence from a Survey of Unlawful File‐Sharing Behavior

**DOI:** 10.1111/risa.12689

**Published:** 2016-09-13

**Authors:** Steven J. Watson, Daniel J. Zizzo, Piers Fleming

**Affiliations:** ^1^ Department of Psychology Lancaster University Lancaster UK; ^2^ Newcastle University Business School Newcastle Upon Tyne UK; ^3^ School of Psychology University of East Anglia Norwich UK

**Keywords:** Affect heuristic, anonymity, trust

## Abstract

Increasing the perception of legal risk via publicized litigation and lobbying for copyright law enforcement has had limited success in reducing unlawful content sharing by the public. We consider the extent to which engaging in file sharing online is motivated by the perceived benefits of this activity as opposed to perceived legal risks. Moreover, we explore moderators of the relationship between perceived risk and perceived benefits; namely, trust in industry and legal regulators, and perceived online anonymity. We examine these questions via a large two‐part survey of consumers of music (*n* = 658) and eBooks (*n* = 737). We find that perceptions of benefit, but not of legal risk, predict stated file‐sharing behavior. An affect heuristic is employed: as perceived benefit increases, perceived risk falls. This relationship is increased under high regulator and industry trust (which actually increases perceived risk in this study) and low anonymity (which also increases perceived risk). We propose that, given the limited impact of perceived legal risk upon unlawful downloading, it would be better for the media industries to target enhancing the perceived benefit and availability of lawful alternatives.

## 1. INTRODUCTION

Most people do not perceive themselves to be lawbreakers, yet downloading music, TV, movies, eBooks, and other media unlawfully is a phenomenally widespread activity. Up to one in six online users report consuming at least some unlawful content online,^1^ and peer‐to‐peer (p2p) file‐sharing networks account for up to a third of all Internet traffic.^2^ This rampant unlawful activity is said to have resulted in extensive harm to the creative industries,^3^, ^4^ to the extent that it is seen as an existential threat to their survival. To stifle these perceived harms, stakeholders have focused on increasing the perceived risk of unlawful file sharing (UFS) by pursuing high‐profile legal cases. However, perceived benefit is likely to be of equal or more importance. We explore the extent to which perceived benefit matters relative to perceived risk in predicting engagement in this widespread yet unlawful behavior. We also consider factors that may impact on the relationship between perceived benefit and perceived risk—the affect heuristic—for UFS behavior; namely, trust in industry and legal regulators, and perceived anonymity online.

### 1.1. Legal Risk and UFS Behavior

If the negative consequences of engaging in an action become more likely or more severe, then people should be less likely to engage in the behavior. There is evidence to suggest that increasing perception of legal risk has appeared to have some effect upon UFS. When the Recording Industry Association of America (RIAA) announced lawsuits would be initiated against individual file sharers, the number of files uploaded for sharing reduced.^5^ Similarly, introducing new legislation may reduce UFS and increase legal sales.^6^ However, targeting risk perception may have limited impact. A nontotal reduction in uploaders has a relatively small impact on UFS, only a few uploaders are required to permit widespread downloading. Also, observed general deterrent effects may be temporary. The reductions in downloading following the announcement of lawsuits contrast with an actual increase in UFS once lawsuits started and users realized the risk was not as bad as anticipated.^7^ Finally, empirical articles often only note a shift in peer‐to‐peer (p2p) downloading activity following the introduction of laws; this may fail to identify users who move to other sources of unlawful content rather than to legal channels.^8^ For example, the introduction of a new law in New Zealand did result in an observed net decrease in total UFS, but also a significant shift away from p2p into alternative methods of UFS.^9^ Overall, although increasing legal risk does appear to moderately reduce UFS^5^ and increase legal sales^6^ it has failed to deter a large number of users from engaging in UFS and the activity remains widespread.

### 1.2. The Benefit of UFS as a Motivating Factor

Entertainment is an emotional medium. Presumably, people engage in UFS because it confers certain benefits. A large‐scale review identified that many motives for engaging in UFS are related to the advantages of UFS compared to legal purchases in terms of price, availability of niche content, ease of access, and flexibility of use.^10^ Many behaviors are more readily predicted by their capacity to deliver pleasurable experience rather than their level of risk.^11^ This is especially true for behaviors engaged in for the purpose of receiving pleasure, such as unprotected sex, rather than behaviors for avoiding harm, such as using a seatbelt.^12^ It is also true that successful prosecutions for engaging in UFS are very rare.^8^ Thus, the emotional benefits of accessing desired media may be much more salient than the potentially remote risk of prosecution. Thus, the perceived benefit of engaging in UFS may be a more powerful driver of UFS behavior than perceived risk, presenting a more powerful target for future interventions.

### 1.3. The Affect Heuristic in UFS

If it is true that UFS is engaged in because of the potential pleasure it confers, then it is likely that the affect heuristic will play a role in the decision to engage in UFS.^13^, ^14^ The affect heuristic refers to the observation that perception of risk is negatively correlated with perception of benefits; in reality, risk and benefit are independent of each other. As one increases or decreases, there is no reason why the other must vary and often the highest rewards come with the highest risks.^15^ Consequently, it may be the case that the desire to engage in UFS reduces the perception of the legal risk of doing so.

Two potential moderators of the affect heuristic are trust and anonymity. The unlawful downloading of files from the Internet presents an opportunity to explore these moderators in a theoretically unique environment when compared to previous research.

### 1.4. Trust in UFS

Trust is one of the most important predictors of risk‐taking behavior.^16^ If we trust a transaction partner to treat us fairly, then we are more likely to engage in risky behaviors with that partner.^17^ However, the role of trust is complicated in UFS by the fact that key relevant partners such as media industries and regulating authorities are responsible for punishing infringers. Thus, the normal relationship whereby higher trust is associated with a reduction in risk perception, and also indirectly with a corresponding increase in perceived benefit via the affect heuristic, may not hold.^18^, ^19^ Instead, higher trust may be associated with greater risk. An additional factor pertinent to UFS is that few individuals will have direct experience of dealing with either the media industry or regulating authorities concerning UFS. Consequently, trust perceptions are likely to reflect general beliefs, possibly informed by beliefs that may reflect the outcome of high‐profile advertising campaigns and litigations made to discourage UFS. When past experience is limited, affective processes can have a larger impact upon trust perceptions.^20^ We can, therefore, anticipate that because people are likely to have limited exposure to regulating authorities and industry with regard to UFS, and because we expect greater trust to be associated with greater risk due to the enforcement role of such organizations, that there will be a stronger affect heuristic under conditions of greater trust, demonstrated by a stronger negative correlation between trust and perceived benefit.^19^

### 1.5. Anonymity in UFS

In comparison to most unlawful activity, engaging in UFS might be perceived as a highly anonymous activity. A huge number of people engage in UFS.^1^, ^2^ Internet users may, therefore, feel “hidden” among a multitude of other users in much the same manner as herding is advantageous for prey animals.^21^, ^22^ Anonymity might be associated with a more reflective, less affective basis for perceptions, whereas those who perceive themselves to be less anonymous may also experience risk assessments more affectively, and be led in their perceptions by high‐profile and emotionally arousing individual cases of file sharers being caught and punished.^13^ Therefore, we expect that as perceived anonymity increases, perceived risk will decrease, as perceived anonymity decreases, the affect heuristic will become more pronounced and perceived risk will increase.

### 1.6. Differences Between Media

The reasons for reading a book are unlikely to be the same as for listening to music. It is therefore no great surprise that the determinants of UFS also appear to differ depending upon media type.^10^ Risk perceptions also differ according to context.^23^ In the case of music there have been high‐profile campaigns to punish infringers. In comparison, the mass digitization of books has been a relatively recent phenomenon with fewer high‐profile legal disputes. Thus, it might be expected that music UFS is considered more risky than the equivalent behavior for eBooks, especially given that highly arousing case studies can have a greater impact on decision making than presentations of facts.^13^ Alternatively, if more experience in UFS leads to lower risk perception and less emotional engagement, then downloading of eBooks will likely be considered the more risky activity.

## 2. METHOD

### 2.1. Participants

Email invitations were sent to a representative U.K. sample via a market research company for participation in a two‐part survey.

Participants were randomly allocated to one of two media types: eBooks (*N* = 1,036, 406 men, 646 women, aged 16–84, *M* = 46.3 years, *SD* = 15.57 years) or music files (*N* = 959, 397 men, 557 women aged 16–82, *M* = 45.0 years, *SD* = 15.80 years). A total of 5,198 participants attempted part one (56% response rate); 2,904 failed to complete, 101 withdrew, 110 were excluded for completing the questionnaire in less than 6 minutes, and 88 were removed for inconsistent demographic data between part one and part two, resulting in a sample of 1,036 + 959 = 1,995 participants.^1^ Two months later, invitations were sent for part two, which added the variable of reported behavior. A total of 1,543 participants also attempted part two (74% response rate). Out of 1,543 participants, the same 88 participants were removed for inconsistent demographic data between part one and part two, 41 failed to complete, and 19 participants withdrew, resulting in a sample of 1,395 participants who completed both parts. This is split between 737 participants for eBooks (309 men, 396 women, aged 16–84, *M* = 47.2 years, *SD* = 15.35 years) and 658 participants for music files (286 men, 346 women, aged 16–83, *M* = 47.3 years, *SD* = 15.36 years).

### 2.2. Materials and Procedure

The eBooks and music file‐sharing questionnaires were identical except that all references to eBooks were replaced with music files. Part one was a multi‐item online questionnaire including questions related to how much risk participants perceived was associated with file sharing, how beneficial participants perceived file sharing to be, and the proposed moderators of the anticipated affect heuristic: trust and anonymity. Median time to complete was 15 minutes. After two months participants completed part two in which they self‐reported file sharing since part one and further questions as part of a separate study. Median time to complete part two was 7 minutes.

#### 2.2.1. UFS Behavior

To estimate engagement in UFS, two items were combined to calculate file‐sharing behavior in the part two questionnaire. First, participants were asked “How many eBooks/music files have you downloaded in the past two months (of all kinds)?” (i.e., since part one), and then they were asked, “What percentage of those eBooks/music files were lawful?” The second score was transformed to calculate the unlawful remainder from 100% and then multiplied by the total number of downloads to calculate the total number of unlawful downloads. The total number of downloads was very heavily skewed, even if log transformed. Therefore, UFS behavior was categorized based on a median split of the nonzero data producing three ordinal categories: zero downloading (music *n* = 540; eBooks *n* = 644), infrequent downloading (up to and including three files; music *n* = 43; eBooks *n* = 57), and frequent downloading (more than three files; music *n* = 75; eBooks *n* = 36). This means that downloading was fairly common in our samples, with 21.9% of respondents engaged in UFS of music and 14.6% of respondents engaging in UFS of eBooks. These estimates are broadly similar to the UFS rates detected in a study by Ofcom (26% for music and 9% for eBooks) when their sample, like ours, is limited to those who consume digital media online.^1^ Our principal dependent variable is perceived risk, and this is estimated from the entire sample, not only those who engaged in UFS.

#### 2.2.2. Risk

Risk was assessed using a six‐item Likert‐scale measure. Three items related to the perceived severity of the consequences for being caught engaging in UFS (e.g., If I was caught downloading eBooks/music unlawfully I think I would face a harsh punishment), and three items related to the perceived likelihood of being caught engaging in UFS (e.g., If I downloaded eBooks/music unlawfully the chance of being punished for it seems very low). These and the remaining questions were asked two months prior to the behavior questions. The scale has adequate internal consistency (Cronbach's α_MUSIC_ = 0.72, Cronbach's α_EBOOKS_ = 0.77).

#### 2.2.3. Benefits of UFS

A seven‐item scale assessed perceptions of the benefits of UFS, including perceived advantages related to quality, flexibility of use, and cost (e.g., I think getting books/music for free is a good reason to download eBooks/music files unlawfully). Internal consistency was adequate (Cronbach's α_MUSIC_ = 0.80, Cronbach's α_EBOOKS_ = 0.76).

#### 2.2.4. Trust

Participants’ trust was measured in two domains. Their trust in the music or book publishing industry, and trust in legal regulators. Trust was measured using eight questions that explored perceptions of fairness (e.g., I think that the way book publishing/music companies deal with users of unlawful download sites is fair), openness (e.g., I think that book publishing/music companies make it easy to find out about their policies with regard to unlawful downloading), care (e.g., The book publishing/music companies' with regard to unlawful downloading, are intended to help the public), and competence (e.g., The book publishing/music companies are competent, with regard to unlawful downloading, to help the public).^19^, ^24^ Both scales had adequate internal consistency (Legal regulators: Cronbach's α_MUSIC_ = 0.77, Cronbach's α_EBOOKS_ = 0.72; Industry: Cronbach's α_MUSIC_ = 0.71, Cronbach's α_EBOOKS_ = 0.69).

#### 2.2.5. Anonymity

A five‐item scale measured participants’ perceived anonymity. Two items examined the ability of participants to avoid detection based on Watling *et al*. 25 (e.g., If I wanted to download eBooks/music unlawfully I am able to lower the risk of being caught). Three items estimated the extent to which participants felt anonymous online (e.g., When you are on the Internet you feel free to act in ways you normally would not). Internal consistency was acceptable (Cronbach's α_MUSIC_ = 0.62, Cronbach's α_EBOOKS_ = 0.61).

### 2.3. Data Analysis

An ordered logit regression was utilized to determine whether relationships exist between perceived risk and benefit with UFS. We used zero UFS as the comparison group to infrequent UFS (one to three files) and frequent UFS (three plus files).

To determine whether the affect heuristic is present, OLS regression was utilized with the perceived benefits of UFS predicting perceived risk. To examine the role of the proposed moderators of the affect heuristic, the procedures proposed for testing two‐way moderation interactions in OLS regression described in Dawson^26^ are utilized.^2^ Briefly, the process uses hierarchical OLS regression. Perceived risk was the outcome variable. In the first step, perceived benefit and a proposed moderator are entered into the regression model (model 1). In the second step, perceived benefits, the moderator, and their interaction are entered into the model (model 2). This permits the existence and effect size of any interaction effect to be determined. The effect sizes of interaction terms are presented in terms of *f* ^2^, which is very similar to *R^2^* change but provides the ratio of variance explained due to only the interaction term in OLS regression. *f* ^2^ can be calculated from:

f2=R model 12−R model 221−R model 22.

## 3. RESULTS AND DISCUSSION

A comparison of the perception of risks, benefits, trust, and anonymity between eBooks and music is provided in Table I.^3^ There was a slightly larger perceived benefit to unlawful music downloading compared to eBooks, whereas trust in the book publishing industry was greater than trust in the music industry. Regulating authorities were also perceived as more trustworthy in the context of eBook downloading than music downloading. These initial findings substantiate the premise that media are perceived differently and should be explored separately in the context of UFS.^10^ There was no difference in perceived risk between media, counter to expectations based on users’ knowledge, or experience of legal prosecutions.

**Table I:** Comparison Between Scale Summary Scores for Music and eBooks

		eBooks (*n* = 737)				Music (*n* = 658)					
Scale	Scale Range	Mean	*SD*	Min	Max	Mean	*SD*	Min	Max	*t*	*p*
Risks	6–42	23.57	6.31	6	42	24.07	6.23	6	42	−1.47	0.142
Benefits	7–49	21.49	7.17	7	47	22.59	8.10	7	47	−2.67^a^	0.008*
Trust in industry	8–56	33.54	6.64	8	56	31.80	7.70	8	56	4.49^a^	<0.001*
Trust in regulating authorities	8–56	33.84	6.83	8	56	32.80	7.23	8	56	2.77	0.006*
Anonymity	5–35	15.44	5.06	5	32	15.37	5.12	5	35	0.261	0.794

^*^ *p* < 0.05.

^a^Equal variances not assumed.

### 3.1. Risk, Benefits, and UFS

The relationship between perceived risk and benefit and reported UFS is illustrated in Table II. An increase in legal risk for UFS was not associated with any statistically significant decrease in self‐reported UFS for either eBooks or music. However, the perceived benefits of UFS did significantly predict increased self‐reported UFS behavior for both eBooks and music. Practically, this suggests a fruitful route to competing with UFS is to provide services that meet the demands of consumers that UFS fulfills. Moreover, it may call into question the legally‐focused media industry strategy where impact on behavior may be limited.

**Table II:** Ordinal Logit Regressions of Perceived Risk and Benefit of UFS on Reported UFS Behavior

Media	Variable	OR	Lower 95% CI	Upper 95% CI	Wald *χ^2^* (1df)	*p*
EBooks	Risk	1.01	0.97	1.05	0.19	0.666
	Benefits	1.07	1.04	1.11	20.43	<0.001*
Music	Risk	1.00	0.96	1.04	0.002	0.965
	Benefits	1.15	1.11	1.18	82.31	<0.001*

^*^ *p* < 0.05.

These findings support evidence that the impacts of legal changes may be short lived or limited.^7^, ^9^ That we did not find any evidence for an effect of legal risk need not necessarily be in complete contradiction to previous studies finding an effect, such as those by Bhattacharjee *et al*.^5^ or Danaher *et al*.^6^ We use a survey sampling approach whereas Bhattacharjee *et al*.^5^ take data directly from a large p2p website and Danaher *et al*.^6^ take their data from iTunes sales data. Thus, the latter studies have much larger samples. It seems plausible that legal risk may have a role to play in UFS, but that the effect is sufficiently small that it can only be observed in extremely large samples. We do not therefore claim that changes to legal frameworks make no difference to consumer behavior, but only that if such effects are present they are only observable at the population level; given that we observe a much more powerful predictor of behavior in perceived benefit, changes to legal frameworks may not be the most effective route to behavior change. Specifically, one strategy to combat UFS would be to provide easy access to information about the benefits of legal purchases or services, in an environment in which the specific benefits UFS confers are met by these legal alternatives. Indeed, the strategy of giving consumers a compelling alternative to UFS has seen Spotify attain 15 million subscribers at the start of 2015, having been launched in October 2008,^27^ and Apple generate revenue of over $16 billion in 2013 via its iTunes service.^28^ The success of these services has partly been obtained by providing benefits to consumers that previously could only easily be obtained via UFS; these include rapid access to a very wide catalogue of content, and the capacity to selectively consume created content. That is, consumers no longer need to buy entire albums if they desire access to only individual songs. These observations support theoretical arguments that it is possible to compete with the UFS market by meeting the needs of consumers.^29^ Moreover, there is evidence suggesting that the development of increasingly appealing legal alternatives to UFS has been the most significant factor in the recent decline of UFS.^30^

### 3.2. The Affect Heuristic and UFS

Risk and benefit ratings correlate negatively for both music (*r* = −0.153, *p* < 0.001) and eBooks (*r* = −0.202, *p* < 0.001). This represents a fairly strong effect of perceived benefit upon perceived risk for UFS.^31^ Finucane *et al*.^31^ assessed the strength of the affect heuristic across a wide range of behaviors and found an average correlation (range) of *r* = −0.12 (0.07 to −0.44). The results of OLS regressions assessing the strength of this relationship in UFS are shown in Table III and demonstrate that perceived risk can be predicted from perceived benefit. This confirms that the perceived benefit of UFS both motivates behavior and, to some extent, undermines the perception of legal risk.

**Table III:** OLS Regressions of Perceived Benefit of UFS on Perceived Risk of UFS

Media	Variable	β	*SE*	*t*	*p*	*R* ^2^
eBooks	Constant	27.41	0.61	45.12	<0.001*	
	Benefits	−0.18	0.03	−6.63	<0.001*	0.04
Music	Constant	26.70	0.58	46.31	<0.001*	
	Benefits	−0.11	0.02	−4.79	<0.001*	0.02

^*^ *p* < 0.05.

### 3.3. Perceived Moderators of the UFS Affect Heuristic

**Figure 1:**
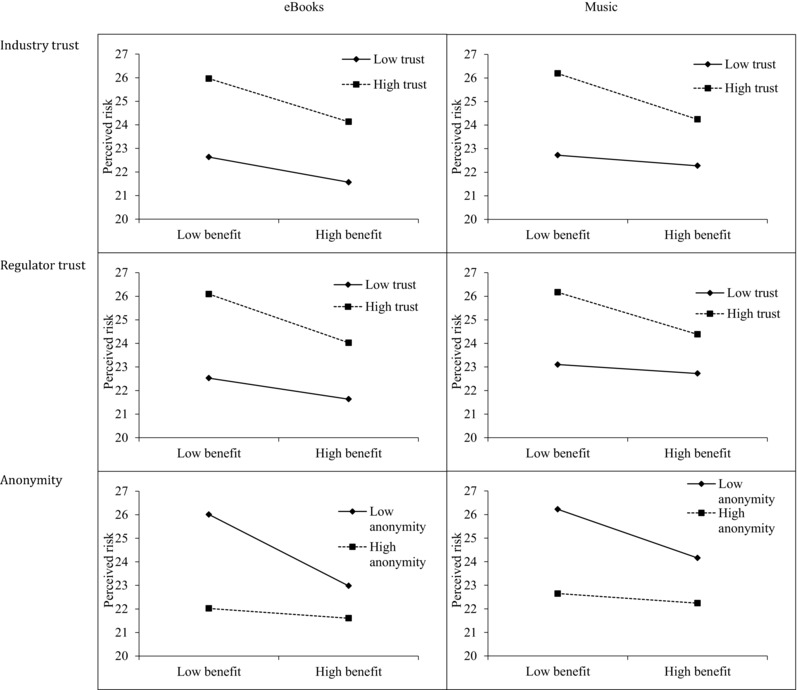
Plots of simple slopes of interaction terms for eBooks (left) and music (right). The interaction terms for eBooks trust in industry and trust in regulators are not statistically significant (*p* > 0.05).

All moderation models are presented in Table IV,^4^ with interaction effects illustrated in Fig. 1. We followed up these analyses with tests of simple slopes to accompany the illustrations in Fig. 1. We provide these in Table V.

**Table IV:** Moderation of Trust and Anonymity on the Affect Heuristic in UFS

Moderator, Media	Model	Variable	β	*SE*	*t*	*p*	*p*	*F*	R^2^	*R* ^2^ Change	*p*	*f* ^2^
Trust in industry												
eBooks	1	Constant	18.27	1.67	11.02	<0.001	35.92	<0.001	0.089			
		Benefits	−0.10	0.03	−3.00	0.003						
		Trust	0.22	0.04	6.12	<0.001						
	2	Constant	15.42	3.05	5.05	<0.001	24.37	<0.001	0.091	0.002	.265	.002
		Benefit	0.03	0.13	0.27	0.790						
		Trust	0.31	0.09	3.63	<0.001						
		Benefit*Trust	−0.00	0.00	−1.12	0.265						
Music	1	Constant	19.66	1.49	13.19	<0.001	24.82	<0.001	0.070			
		Benefits	−0.06	0.03	−1.95	0.051						
		Trust	0.18	0.03	5.52	<0.001						
	2	Constant	15.60	2.54	6.15	<0.001	17.92	<0.001	0.076	0.006	.049*	.006
		Benefit	0.12	0.010	1.23	0.221						
		Trust	0.31	0.07	4.24	< .001						
		Benefit*Trust	−0.01	0.00	−1.98	0.049						
Trust in regulators												
eBooks	1	Constant	18.36	1.63	11.26	<0.001	36.31	<0.001	0.090			
		Benefits	−0.10	0.03	−3.02	0.003						
		Trust	0.22	0.04	6.19	<0.001						
	2	Constant	14.04	3.09	4.55	<0.001	25.17	<0.001	0.093	0.003	.100	.003
		Benefit	0.10	0.13	0.79	0.430						
		Trust	0.35	0.09	4.04	<0.001						
		Benefit*Trust	−0.01	0.00	−1.65	0.100						
Music	1	Constant	20.30	1.65	12.28	<0.001	19.26	<0.001	0.056			
		Benefits	−0.07	0.03	−2.05	0.041						
		Trust	0.16	0.04	4.43	<0.001						
	2	Constant	15.80	2.75	5.75	<0.001	14.30	<0.001	0.062	0.006	.041*	.006
		Benefit	0.13	0.10	1.29	0.198						
		Trust	0.30	0.08	3.90	<0.001						
		Benefit*Trust	−0.01	0.00	−2.05	0.041						
Perceived anonymity												
eBooks	1	Constant	29.69	0.82	36.13	<0.001	30.90	<0.001	0.078			
		Benefits	−0.10	0.04	−2.73	0.006						
		Anonymity	−0.26	0.05	−5.28	<0.001						
	2	Constant	35.80	2.10	17.06	<0.001	24.18	<0.001	0.090	0.012	.002*	.013
		Benefit	−0.40	0.10	−3.91	<0.001						
		Anonymity	−0.65	0.13	−4.91	<0.001						
		Benefit*Anonymity	0.02	0.01	3.16	0.002						
Music	1	Constant	29.43	0.85	34.64	<0.001	22.73	<0.001	0.065			
		Benefits	−0.06	0.03	−1.96	0.051						
		Anonymity	−0.26	0.05	−5.13	<0.001						
	2	Constant	33.13	1.95	17.01	<0.001	16.72	<0.001	0.071	0.006	.035*	.006
		Benefit	−0.23	0.09	−2.69	0.007						
		Anonymity	−0.49	0.12	−4.03	<0.001						
		Benefit*Anonymity	0.01	0.01	2.12	0.035						

Outcome variable in all cases is perceived risk of UFS.

^*^ *p* < 0.05.

**Table V:** Simple slopes illustrating the moderating effect of trust and anonymity upon the relationship between perception of risk and benefit

Moderator	Media	Moderator level	Moderator value	Beta	*t*	*p*
Trust in industry	eBooks	Low trust	26.9	‐0.08	‐1.97	0.05*
		High trust	40.18	‐0.13	‐3.01	0.003*
	Music	Low trust	24.11	‐0.03	‐0.69	0.489
		High trust	39.5	‐0.12	‐2.77	0.005*
Trust in regulator	eBooks	Low trust	27.02	‐0.07	‐1.68	0.094
		High trust	40.67	‐0.15	‐3.35	0.001*
	Music	Low trust	25.58	‐0.03	‐0.83	0.41
		High trust	40.03	‐0.12	‐2.89	0.004*
Anonymity	eBooks	Low anonymity	10.38	‐0.21	‐4.18	<.001*
		High anonymity	20.51	‐0.02	‐0.52	0.601
	Music	Low anonymity	10.25	‐0.13	‐2.88	0.004*
		High anonymity	20.49	‐0.02	‐0.55	0.583

a ^*^ *p* < .05

#### 3.3.1. Trust

Higher trust in industry and regulators was associated with greater perceived risk. Greater trust is usually associated with a lowered perception of risk.^17^ However, we find that the role of trust is context specific and high trust in potentially malevolent forces may lead to an enhanced rather than diminished sense of risk.

That said, trust in industry and trust in legal regulators were identified as moderators of the affect heuristic in music UFS (*p* < 0.05) and trust in regulators may be a moderator of the affect heuristic in eBooks (*p* = 0.1). In all these cases, when trust was higher, perceived benefit reduced perceived risk (and vice versa) to a greater extent. Trust in industry did not act as a moderator in eBooks.

In general, the strength of the affect heuristic was enhanced when trust was high, although the evidence for this is stronger in music than eBooks. The simple slopes analysis presented in Table V shows that, when trust is low in the music industry or regulating authorities, the affect heuristic is actually no longer present for UFS of music.

Previous work has shown the importance of trust in the risk–benefit association, although the evidence to date has been in the context of increased trust being associated with decreased risk perception and therefore unlike our findings. However, the proposed mechanism for the trust–affect association from past work is not contradicted by our findings. Trust refers to a willingness to put oneself in a vulnerable position before another party. If trust in that other party is low, one is less likely to simply accept the assessment of risk of that other party, and one must instead consider the likelihood of negative consequences with greater care.^32^ That is, when an institution or individual is not trusted we might be more suspicious and make a more considered assessment of risk and benefit. Those who are more suspicious of the role of regulators and industry might think more carefully about the consequences of file sharing, even if they ultimately conclude it is less risky. In such scenarios judgments will be less emotionally driven and so the affect heuristic will operate less, or even not at all. Conversely, those who trust industry and regulators would believe in their competence. This would be associated with a greater use of the affect heuristic. A related alternative explanation may be that this finding reflects *post hoc* justification. People who express high trust in regulating authorities may have greater fear for the consequences of engaging in UFS as they believe the consequences is more likely. This increased affective response may influence their use of the affect heuristic, particularly in cases such as UFS, where the limited past experience of consumers with regulating and authorities permits a greater influence for affective processes.^20^

Practically, our findings suggest that it may be possible to diminish the perceived benefit of UFS by increasing risk perception, but only to the extent that UFS is considered affectively, and users trust industry and regulators. Increasing trust in industry and regulators may be one route toward encouraging UFS to be considered in affective rather than rational terms. However, given the limited impact of risk perception upon behavior, a better strategy would be to provide a desirable legal alternative.

#### 3.3.2. Anonymity

Greater perceived anonymity was associated with lower perceived risk for both eBooks and music (*p* < 0.05). High anonymity was also identified as a moderator of the affect heuristic (*p* < 0.05). Specifically, it reduced the association between perceived benefits and perceived risk. The relationships are as hypothesized and support the view that those who feel anonymous and lost in a crowd while engaging in UFS rely less on emotion‐based judgment when evaluating risk. The simple slopes analyses presented in Table V show that it is only when anonymity is perceived as being low that the affect heuristic operates for UFS of both books and music.

Overall, restricting the perceived level of anonymity available online may lead people to perceive UFS to be a higher risk. Campaigns that advertise that anonymity online is something of a myth might expect to produce only limited benefit when the relative impact of perceived risk and benefit upon behavior is considered. However, that anonymity is a driver of risk perception could be an important theoretical finding for other online behaviors. For example, the use of services that promise enhanced privacy such as the DuckDuckGo search engine or Tor anonymity network may be associated with increased engagement in risky online behavior.

## 4. CONCLUSIONS

There is evidence of use of the affect heuristic in UFS, in that increases in perceived benefit are correlated with reductions in perceived risk. This is particularly true for those who are high in trust and low in perceived anonymity. Two key, novel theoretical findings are that (1) greater trust leads to greater risk perception if the trusted entity causes harm instead of offering security and (2) anonymity, as well as trust, moderates the affect heuristic with reduced evidence of affect with high anonymity.

Despite this, however, it remains clear that UFS is a behavior engaged in for the benefits it confers and so we expect interventions seeking to undermine these perceived benefits and especially those offering legal alternatives to be the most efficacious. This approach should be adopted for UFS particularly, but may have relevance in any realm where the affective benefits of engaging in a crime are more salient than the potential legal risk of capture. Offline examples may include the use of illegal drugs or the unlawful use of sex workers. Given the power of perceived benefit and the low salience of legal risk, it is perhaps no surprise that legal interventions have a limited and possibly short‐term effect, whereas legal services that compete with UFS have attracted significant numbers of consumers.

## Footnotes

1 The questionnaire had 150 questions and excluding participants who on average spent less than 2.4 seconds on each question was a way of removing participants with obviously insufficient attention paid to the task. All participants were randomly allocated to either have their IP address revealed to them or not. However, this manipulation had no identifiable impact upon results and so is not reported here.

2 We also considered an alternative analysis comprising a structural equation model with trust in industry, trust in regulators, and anonymity moderating a proposed relationship between risk and perceived benefits, which both had direct effects upon reported UFS (low, medium, high) using diagonally weighted least squared estimation. However, the ordinal segregation of UFS led to a poorly identified model due to the comparatively small proportion of participants in the frequent and infrequent file‐sharer categories compared to the non‐file‐sharing category. Given the strong division observed between those who engage in no and very little UFS and the long tail of more frequent file sharers, we felt it inappropriate to change the proposed dependent variable and so reverted to the more basic analysis method reported here.

3 For robustness, we also used a model that included sex and age as demographic factors. Sex and age did not predict behavior in eBooks, but males (β = −0.525, *p* = 0.034) and possibly younger participants (β = −0.014, *p* = 0.102) reported engaging in more music UFS. The effect of risk and benefit upon behavior were not impacted by including these additional variables.

4 Again, for robustness we also built models including sex and age. In all analyses, males and younger participants perceived lower risk; however, the reduction in power prevented exploration beyond main effects when these variables were incorporated.
